# Supplementary material for: Apparent nosocomial adaptation of Enterococcus faecalis predates the modern hospital era
Source: Nat Commun. 2021 Mar 9;12:1523. doi: 10.1038/s41467-021-21749-5 (PMC7943827; doi:10.1038/s41467-021-21749-5)
Supplement: Supplementary file 1 — Supplementary information [file 41467_2021_21749_MOESM1_ESM.pdf]

Supplementary Information:

Supplementary Figures 1–16 and Supplementary Table 1

Title: Apparent nosocomial adaptation of *Enterococcus faecalis* predates the modern hospital era

Author information:

Anna K. Pöntinen[1*], Janetta Top[2], Sergio Arredondo-Alonso[1,2], Gerry Tonkin-Hill[3], Ana R. Freitas[4], Carla Novais[4], Rebecca A. Gladstone[1], Maiju Pesonen[5], Rodrigo Meneses[2], Henri Pesonen[1], John A. Lees[6], Dorota Jamrozy[3], Stephen D. Bentley[3], Val Fernandez Lanza[7], Carmen Torres[8], Luisa Peixe[4], Teresa M. Coque[7,9], Julian Parkhill[10,11], Anita C. Schürch[2], Rob J. L. Willems[2], Jukka Corander[1,3,12*]

[*] Corresponding authors: Anna K. Pöntinen (a.k.pontinen@medisin.uio.no), Jukka Corander (jukka.corander@medisin.uio.no)

These authors contributed equally: Anita C. Schürch, Rob J. L. Willems, Jukka Corander

Affiliations:

[1] Department of Biostatistics, Faculty of Medicine, University of Oslo, Oslo, Norway

[2] Department of Medical Microbiology, University Medical Center Utrecht, Utrecht, The Netherlands

[3] Parasites and Microbes, Wellcome Sanger Institute, Cambridge, United Kingdom

[4] UCIBIO/REQUIMTE. Laboratory of Microbiology, Biological Sciences Department, Faculty of Pharmacy, University of Porto, Porto, Portugal

[5] Oslo Centre for Biostatistics and Epidemiology (OCBE), Oslo University Hospital Research Support Services, Oslo, Norway
[6] MRC Centre for Global Infectious Disease Analysis, Department of Infectious Disease Epidemiology, Imperial College London, London, United Kingdom
[7] Department of Microbiology, Ramón y Cajal Institute for Health Research, Ramón y Cajal University Hospital, Madrid, Spain
[8] Department of Food and Agriculture, Area of Biochemistry and Molecular Biology, University of La Rioja, Logroño, Spain
[9] CIBER in Epidemiology and Public Health (CIBERESP), Madrid, Spain
[10] Wellcome Sanger Institute, Cambridge, United Kingdom
[11] Department of Veterinary Medicine, University of Cambridge, Cambridge, United Kingdom
[12] Helsinki Institute of Information Technology, Department of Mathematics and Statistics, University of Helsinki, Helsinki, Finland

a

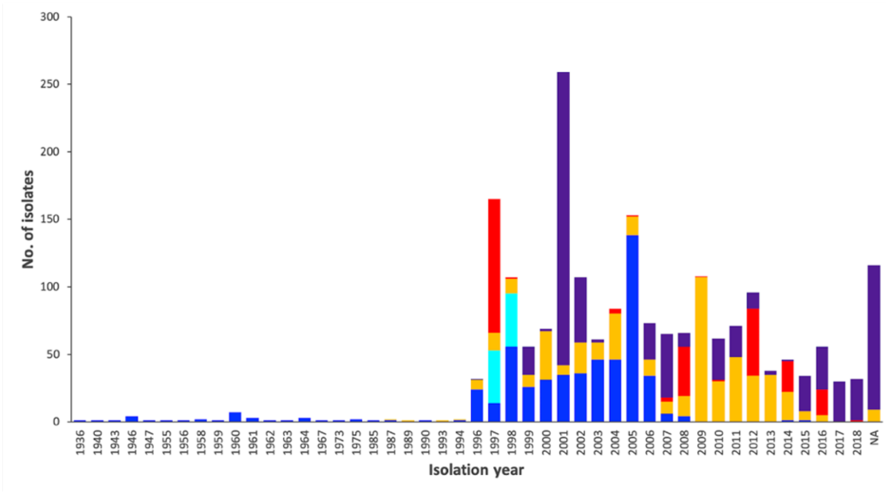

b

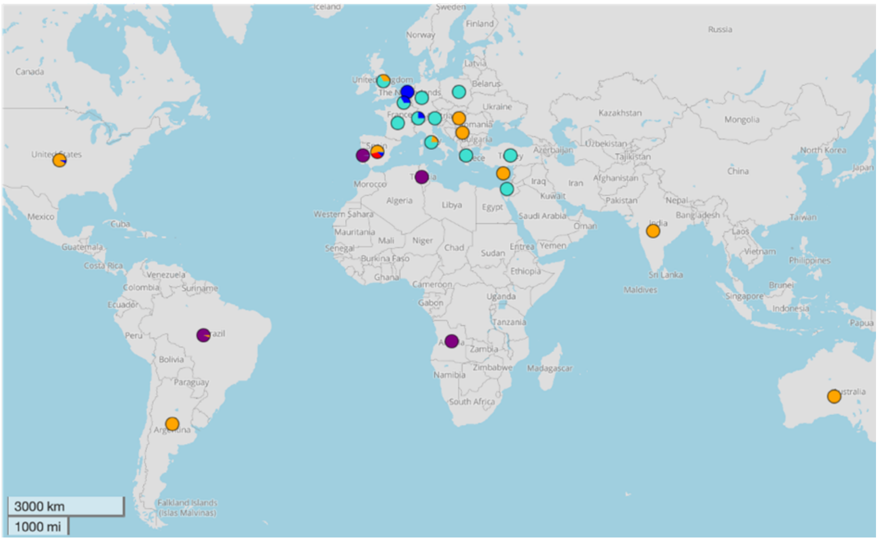

Supplementary Fig. 1: Temporal and geographic distribution of the *E. faecalis* collection.

Total of 2,027 *E. faecalis* isolates were used in the study, representing collections from University Medical Center Utrecht, The Netherlands (*n* = 535; blue), European Network for Antibiotic Resistance and Epidemiology (ENARE) at the University Medical Center Utrecht, The Netherlands (*n* = 78; light blue), University Hospital Ramòn y Cajal, Spain (*n* = 503; orange), University of Porto, Portugal (*n* = 671; purple), and University of La Rioja, Spain (*n* = 240; red). **a,** Number of isolates per isolation years and isolate collections. **b,** Geographic distribution of isolates, created by using Microreact[1]. Source data are provided as a Source Data file.

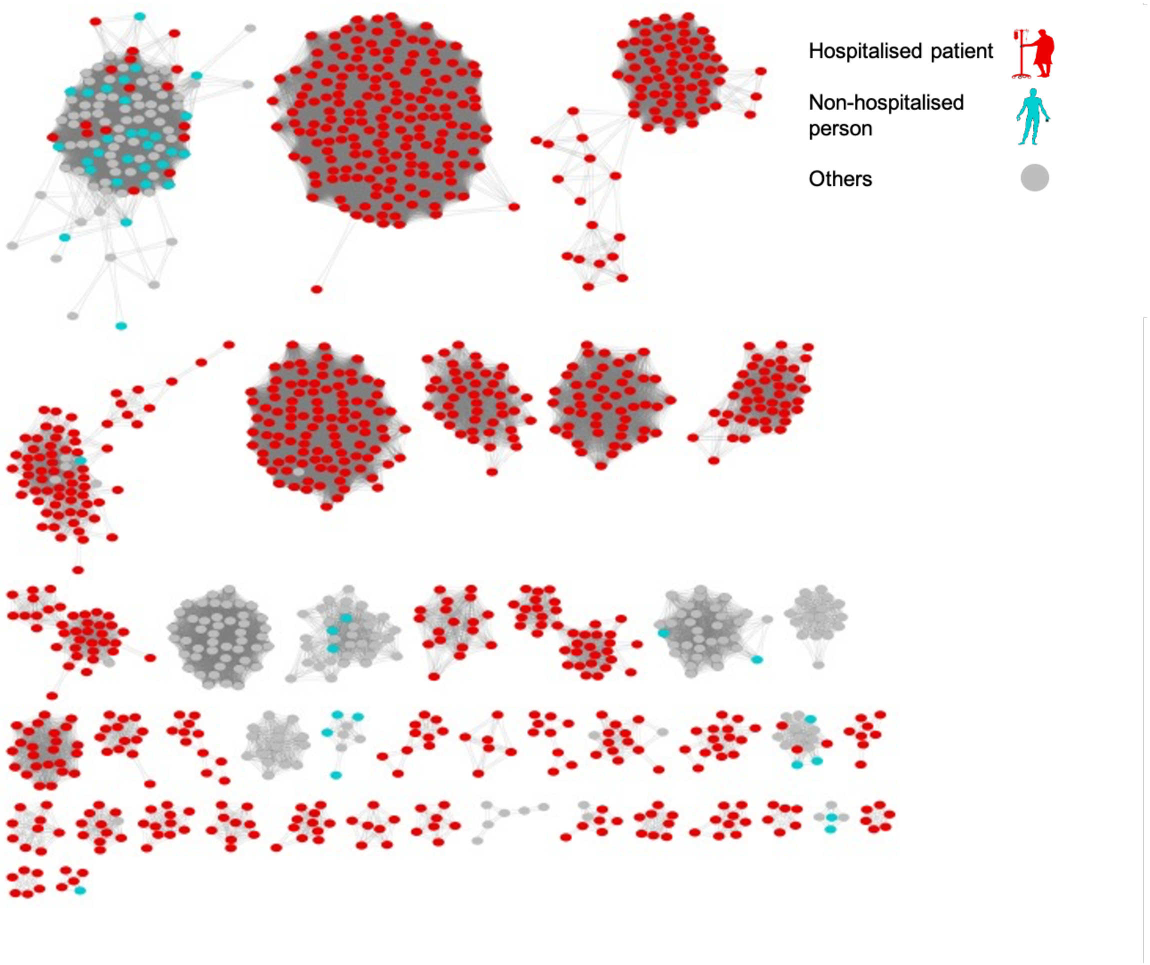

Supplementary Fig. 2: Network analysis of the *E. faecium* accessory genomes, as defined by Panaroo[2], depicts clearly defined hospital-associated (HA) clusters. Nodes indicate separate isolates, connected when shared ≥ 95% of their accessory genome, and colour-coded according to their isolation sources as indicated in the legend: hospitalised patient (red), non-hospitalised person (light blue), and others (grey). Components of less than five isolates were filtered out, and the resulting network was visualised using Cytoscape[3]. Source data are provided as a Source Data file.

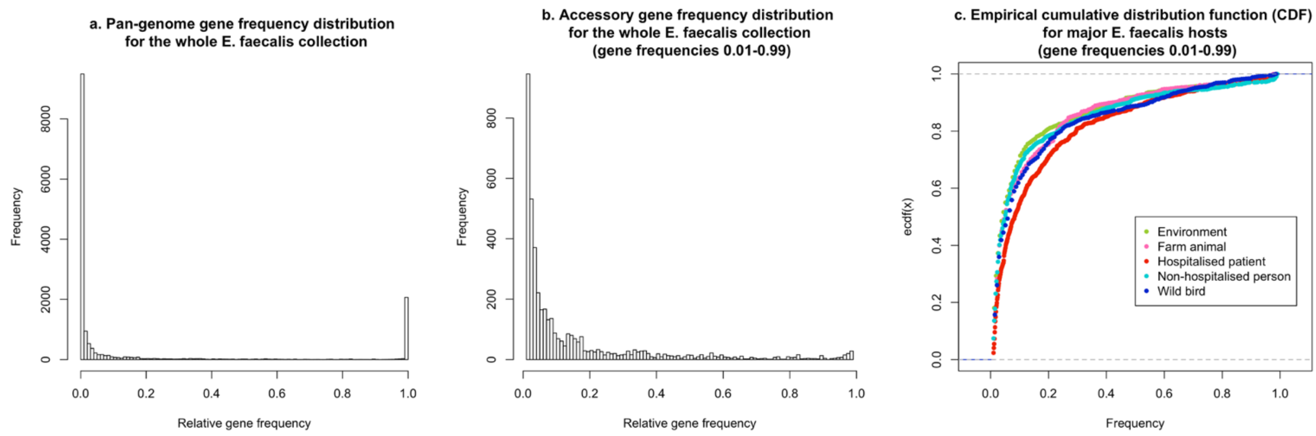

Supplementary Fig. 3: Accessory gene frequency distributions present no significant differences between different host types, indicating generalist nature for *E. faecalis*. a, Pangenome gene frequency histogram for the whole collection of 2,027 *E. faecalis* isolates. **b,** Accessory gene frequency distribution for the whole collection, with gene frequencies of 1–99 %. **c,** Empirical cumulative distribution functions (CDFs) of 1–99% gene frequencies for major *E. faecalis* host types as indicated by colour coding: environment (green), farm animal (pink), hospitalised patient (red), non-hospitalised person (light blue), and wild bird (dark blue). There were no significant differences between empirical CDFs of hospital-associated (HA) isolates and other host types ($P > 0.20$; one-sided permutation tests with the maximum difference of each pair of empirical CDFs as the test statistic).

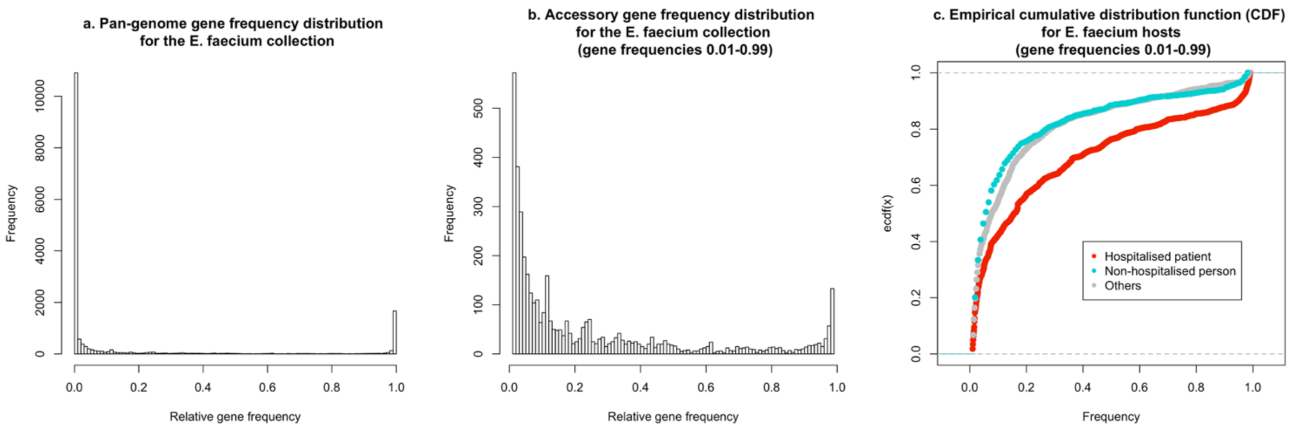

Supplementary Fig. 4: Accessory gene frequency distributions indicate adaptation of *E. faecium* isolates of hospital origin. a, Pangenome gene frequency histogram for the 1,602 *E. faecium* isolates[4]. **b,** Accessory gene frequency distribution, with gene frequencies of 1–99 %. **c,** Empirical cumulative distribution functions (CDFs) of 1–99% gene frequencies for *E. faecium* host types as indicated by colour coding: hospitalised patient (red), non-hospitalised person (light blue), and others (grey). Empirical CDF for hospital-associated (HA) *E. faecium* isolates differed significantly from those of non-hospital origins ($P < 0.05$; one-sided permutation tests with the maximum difference of each pair of empirical CDFs as the test statistic).

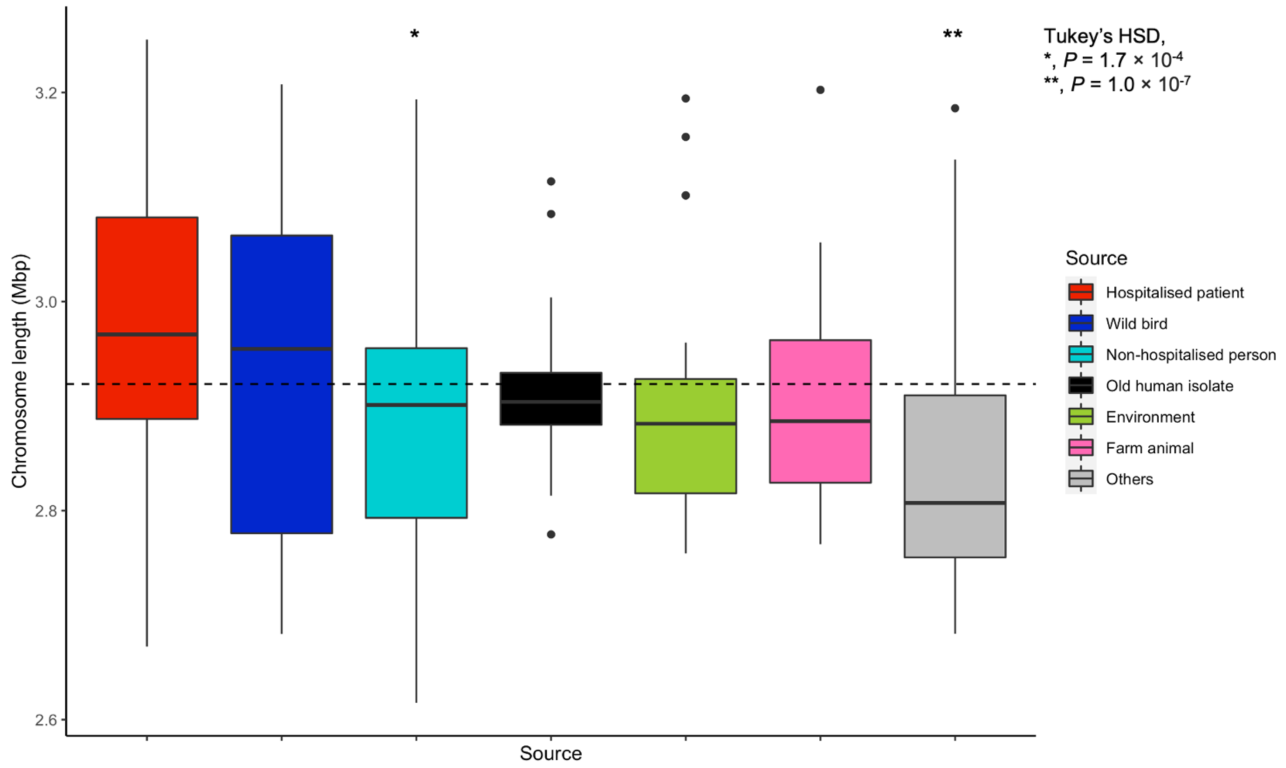

Supplementary Fig. 5: Comparisons of hybrid assembly chromosomes show mainly stable chromosome sizes (Mbp) across different host types in *E. faecalis*, reflecting the generalist lifestyle. Boxplots show the median values of chromosome lengths from isolates colour-coded as per their isolation source: hospitalised patient (red; *n* = 967), wild bird (dark blue; *n* = 136), non-hospitalised person (light blue; *n* = 391), old human isolates (black; *n* = 28), environment (green; *n* =156), farm animals (pink; *n* = 130), and others (grey; *n* = 219). First and third quartiles are shown and whiskers extending at most to the value of 1.5*inter-quartile range from the hinge. Individual dots depict single outliers, and the dashed line indicates mean chromosome length across all sources. *, $P = 1.7 \times 10^{-4}$; **, $P = 1.0 \times 10^{-7}$, as compared to the mean chromosome size of hospitalised patient isolates; Tukey's HSD, accounting for multiple comparison. Source data are provided as a Source Data file.

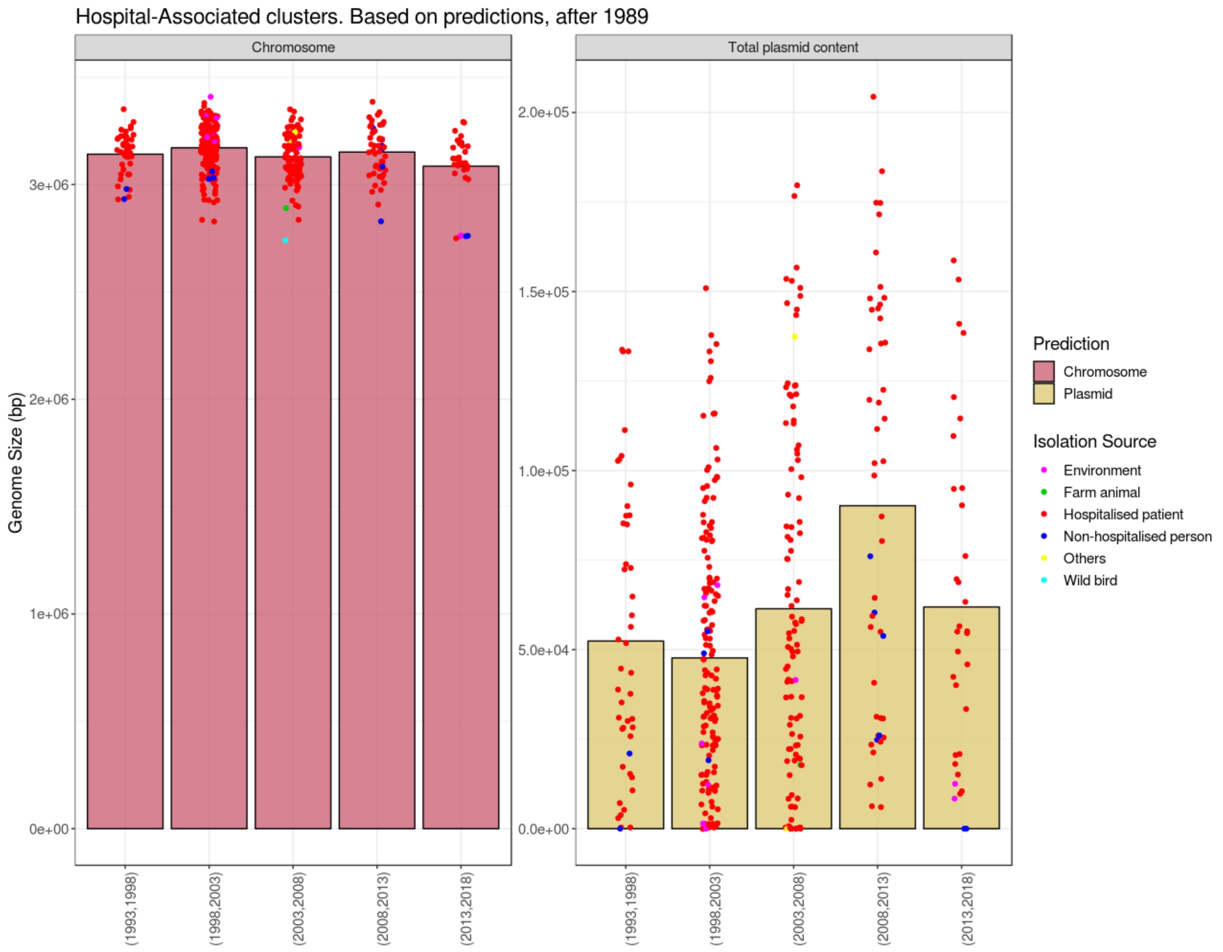

Supplementary Fig. 6: Predicted chromosome (pink) sizes (bp) in the hospital-associated (HA) clusters show no increase over the years of isolation, while a slight intermittently increasing trend is seen in the predicted plasmid content (yellow-green) sizes (bp). Predictions were derived from mlplasmids[5]. Bar plots represent mean genome sizes (bp), and each node represents a single isolate, coded by colour as indicated in the legend: environment (pink), farm animal (green), hospitalised patient (red), non-hospitalised person (dark blue), others (yellow), and wild bird (light blue). Years are shown in intervals of five years.

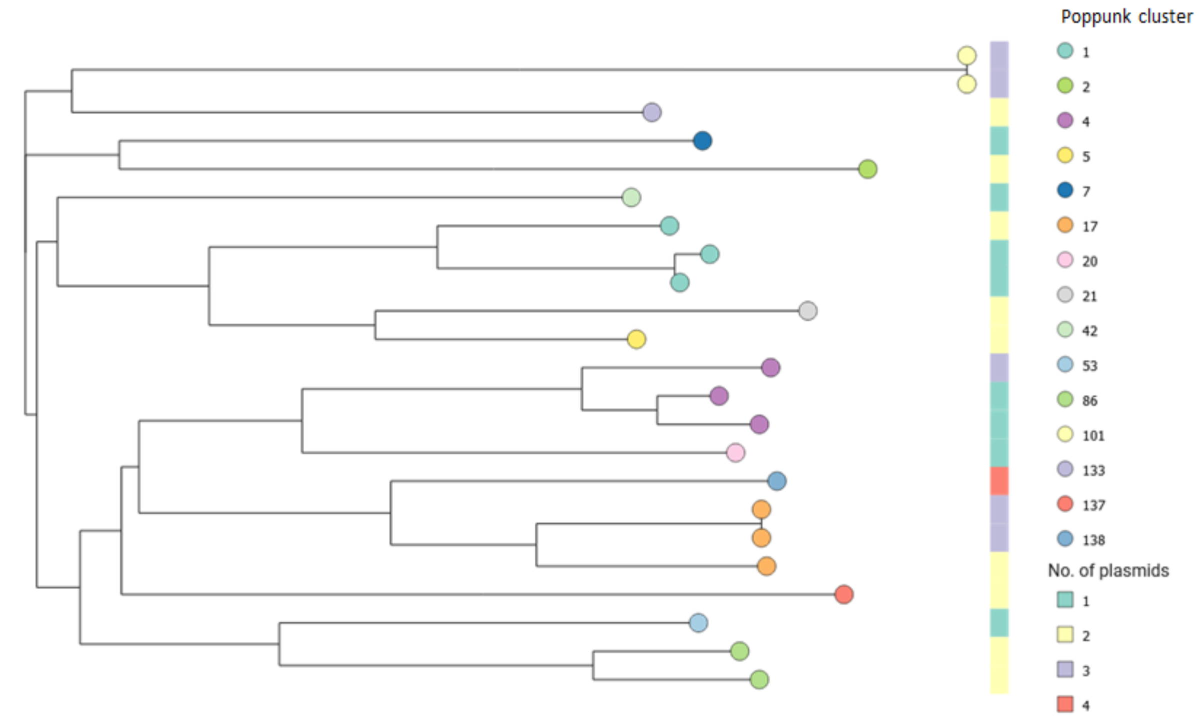

Supplementary Fig. 7: Alignment-free k-mer based clustering[6] of 23 old isolates shows largely diverse genomic background. Nodes are labelled by colour, as indicated in the legend, according to their cluster defined by Population Partitioning Using Nucleotide K-mers (PopPUNK)[7]. The number of plasmids for each strain is coded in the dendrogram by colour as indicated in the legend: 1 (turquoise), 2 (yellow), 3 (purple), and 4 (red).

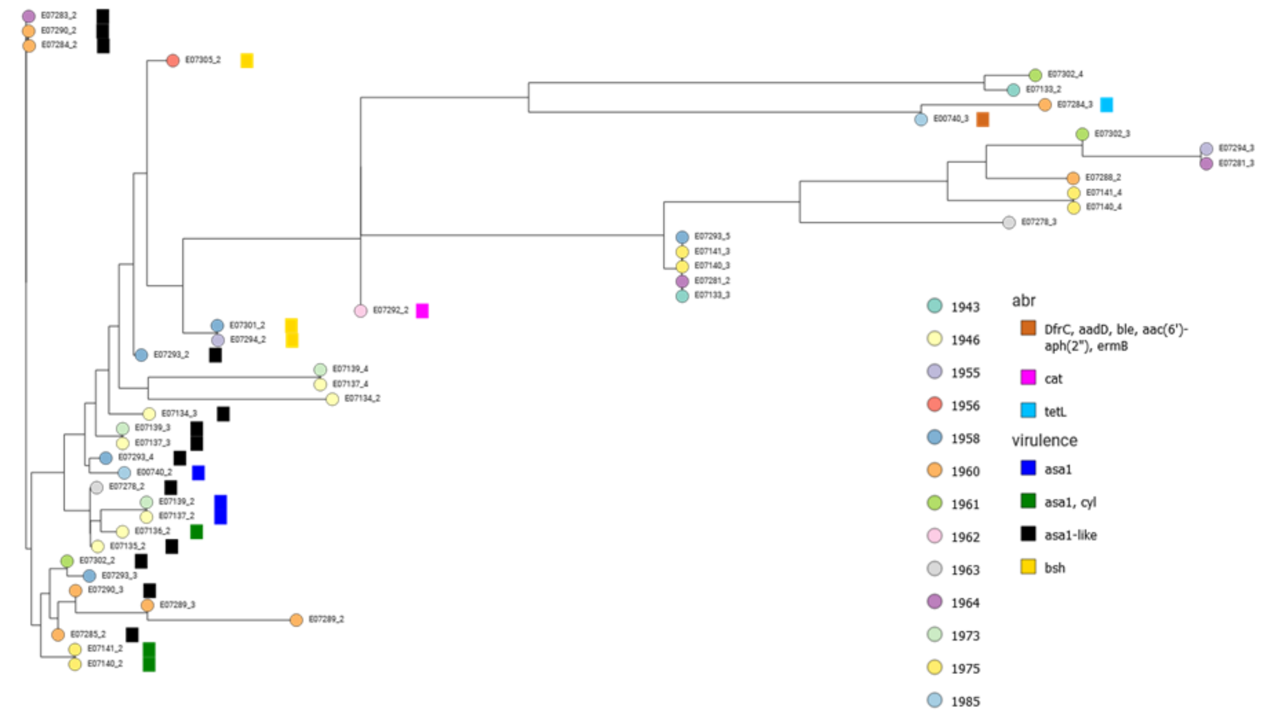

Supplementary Fig. 8: Clustering of presence and absence of k-mers[6] of all 45 identified plasmids among 23 old isolates reveals large plasmid diversity. Nodes are coloured according to isolation year as indicated in the legend. The presence of antibiotic resistance genes (abr) and/or virulence genes are indicated next to the plasmid name, coloured as indicated in the legend.

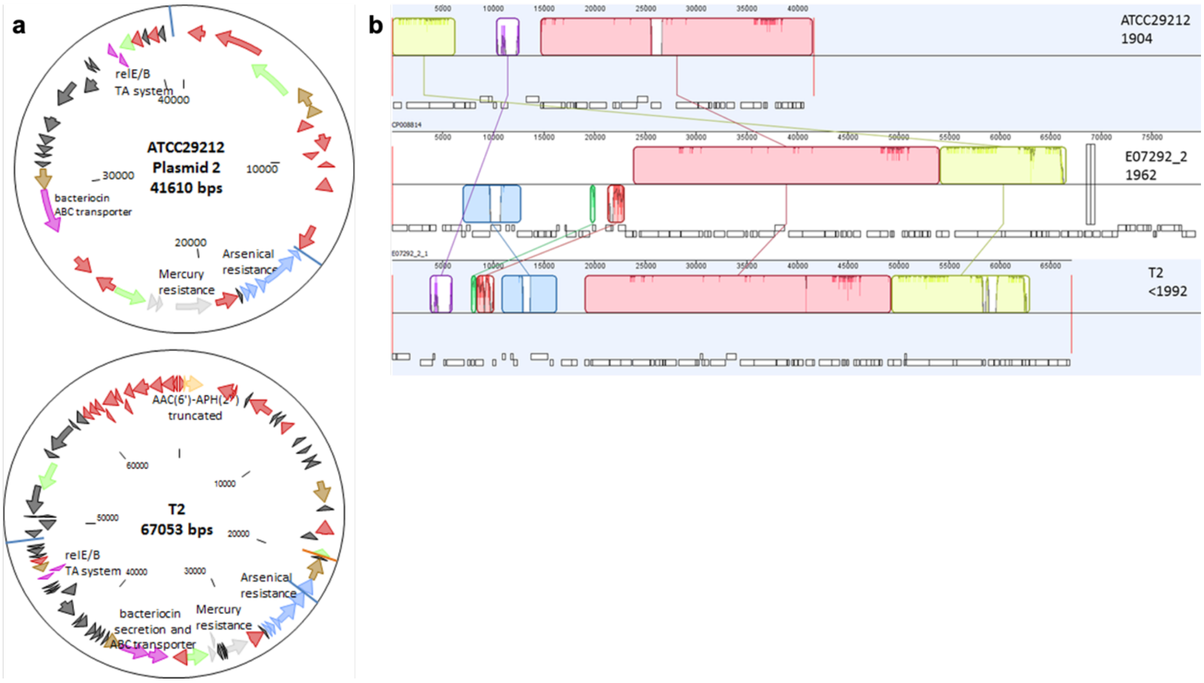

Supplementary Fig. 9: One of the old *E. faecalis* plasmids from 1962 shows identical regions to plasmids geographically and temporally widely distributed. a, Genomic organization for strain ATCC29212 plasmid 2 and the T2 plasmid, isolated in the UK in 1904 and in Japan prior to 1992, respectively. Red arrows indicate plasmid associated genes and transposases, yellow arrows indicate antimicrobial resistance genes, light grey mercury resistance and light blue arsenical resistance genes. **b,** MAUVE alignment[8] of the plasmids from strain ATCC29212, E07292, and T2, indicating identical regions shown in red and yellow.

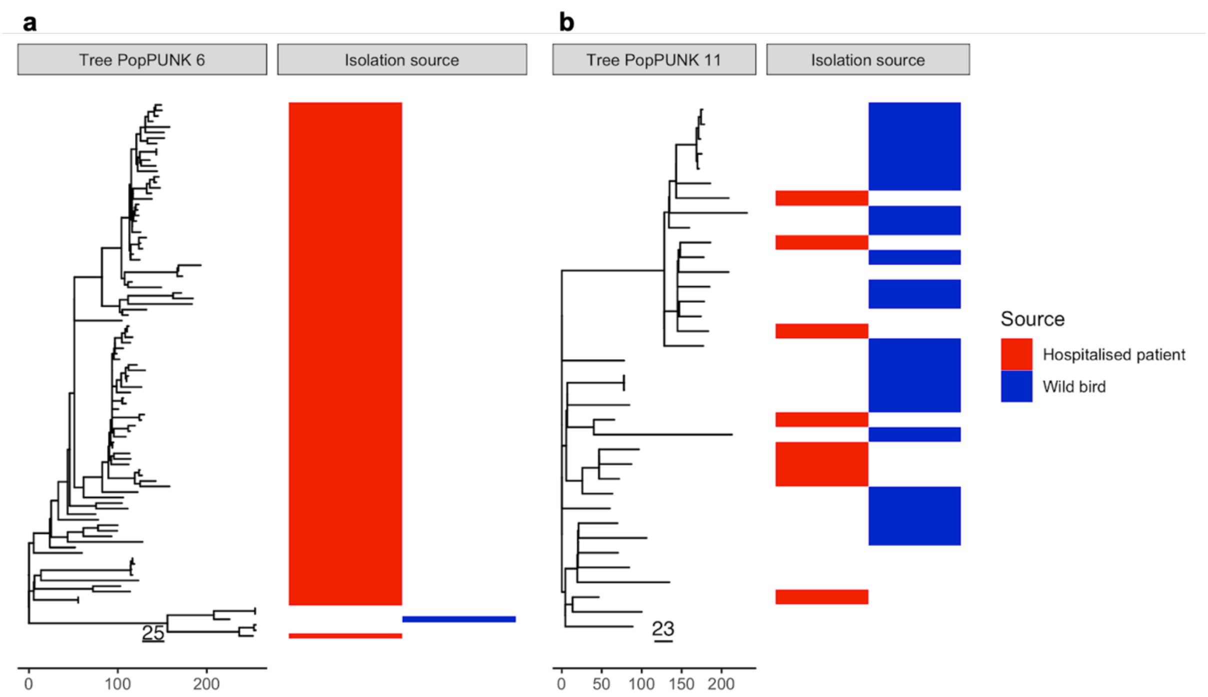

Supplementary Fig. 10: Hospital- and wild bird-associated clusters depict links between the isolation sources within phylogenetic construction. An example of a hospital-associated (HA) Population Partitioning Using Nucleotide K-mers (PopPUNK; PP)[7] cluster PP6 (*n* = 97) (**a**) and cluster PP11 (*n* = 36) (**b**), including isolates of both clinical (red) and wild bird (dark blue) origin, aligned with a maximum-likelihood (ML) cluster phylogeny. Source data are provided as a Source Data file.

a

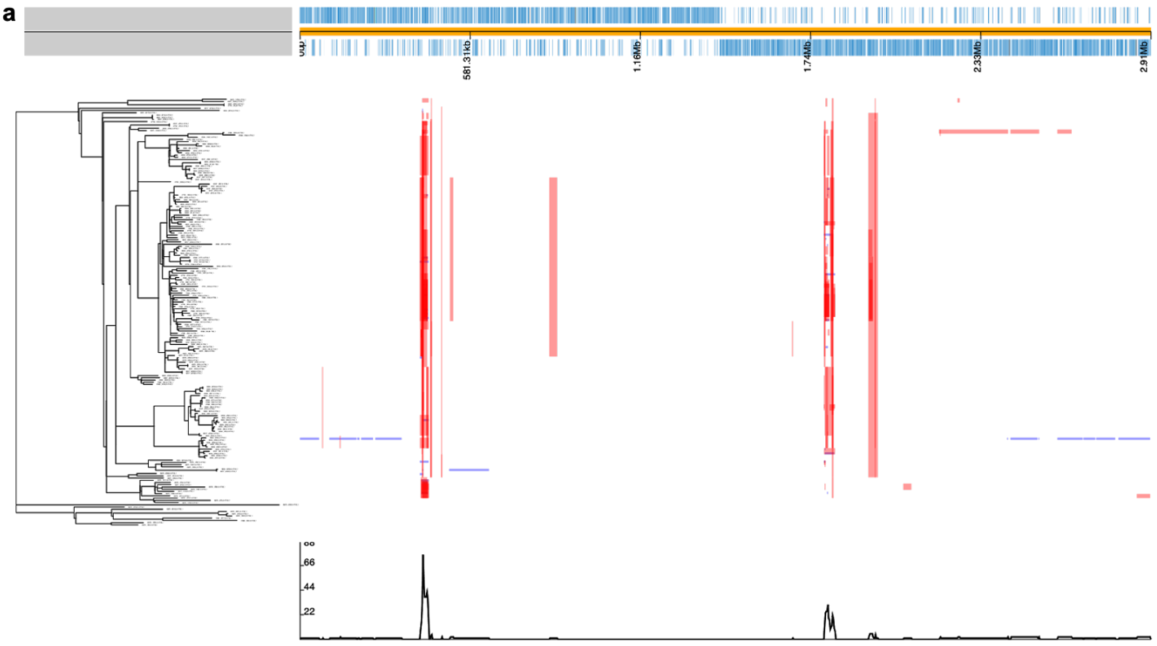
581.31kb
1.16Mb
1.74Mb
2.33Mb
2.91Mb
.66
.44
.22

b

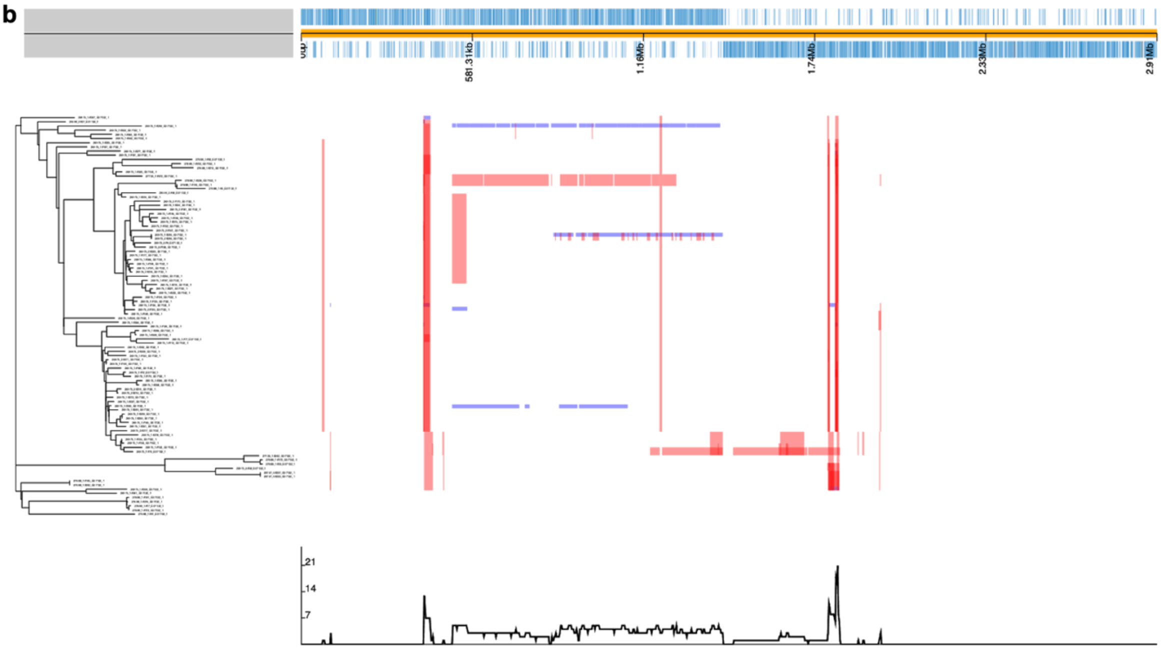
581.31kb
1.16Mb
1.74Mb
2.33Mb
2.91Mb
.21
.14
.7

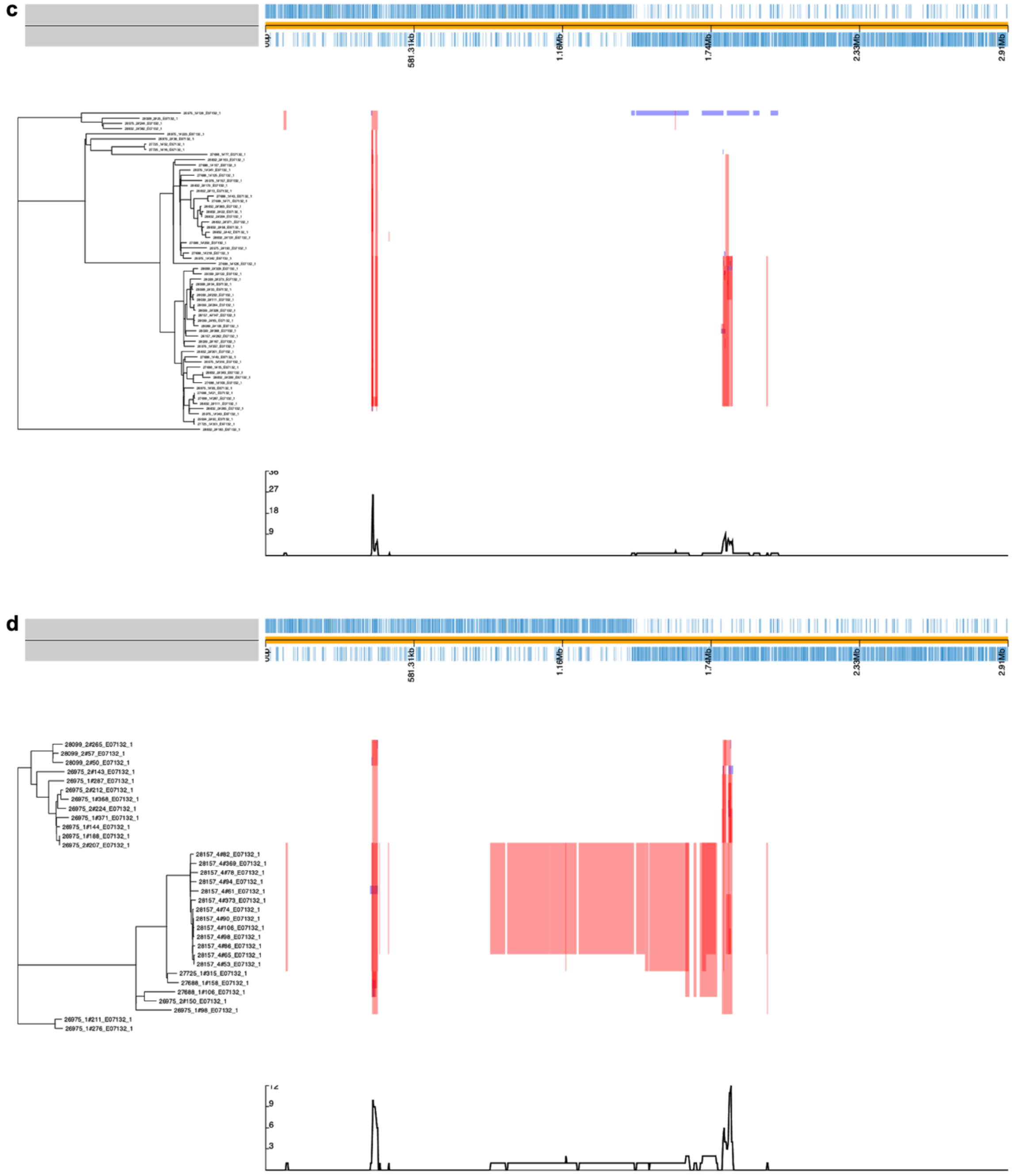

Supplementary Fig. 11: Recombination predictions by using Genealogies Unbiased By recomBinations In Nucleotide Sequences (Gubbins)[9], for hospital-associated (HA) Population Partitioning Using Nucleotide K-mers (PopPUNK)[7] clusters (PP). a, PP2 (*n* = 193). **b,** PP6 (*n* = 97). **c,** PP7 (*n* = 62). **d,** PP18 (*n* = 32). Alignments on the hybrid assembly of *E. faecalis* old isolate E07132. Recombination blocks across maximum-likelihood (ML) cluster phylogeny are depicted by using Phandango[10]: red colouring indicates ancestral blocks

(occurring at a non-terminal node), while blue indicates single isolates only. Reference genome annotation panel above the recombination blocks shows the linearised genome, with genes depicted as blue rectangles. Number of recombination events is plotted underneath the recombination blocks.

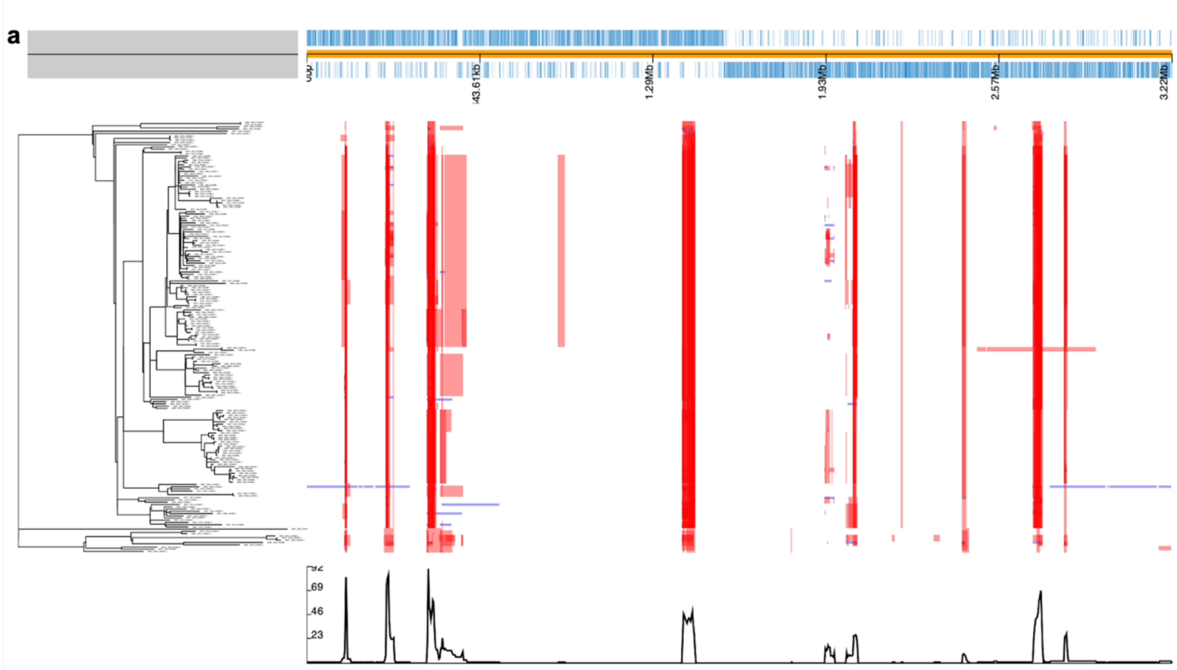
a
43.61kb
1.29Mb
1.93Mb
2.57Mb
3.22Mb
.69
.46
.23

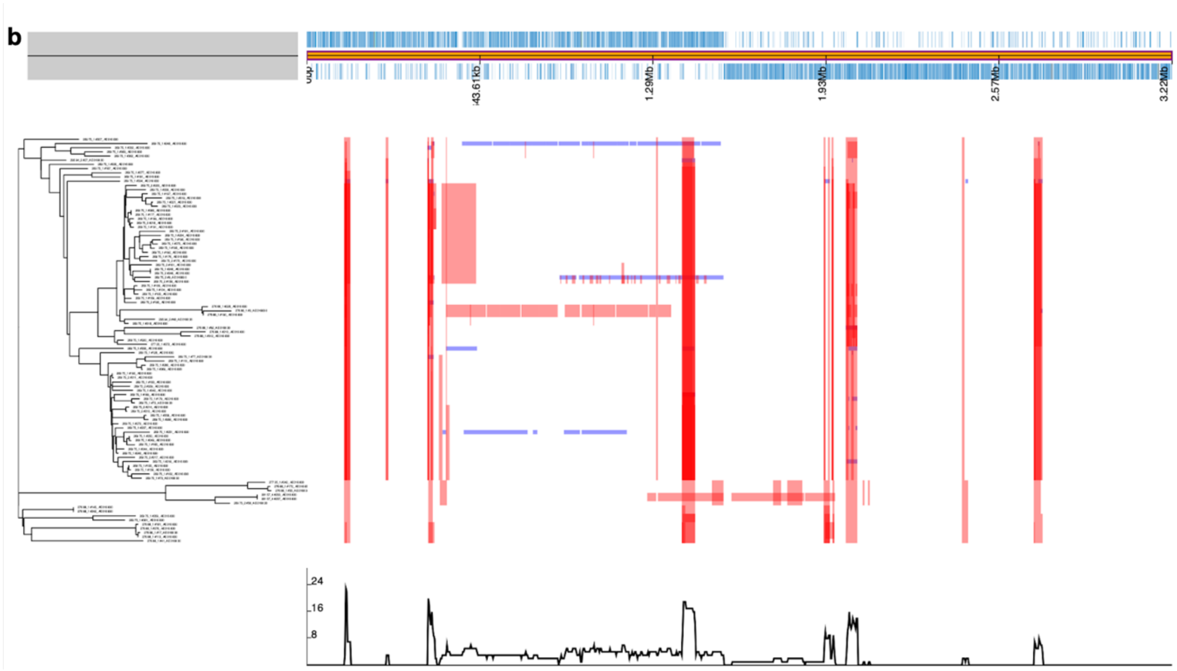
b
43.61kb
1.29Mb
1.93Mb
2.57Mb
3.22Mb
.24
.16
.8

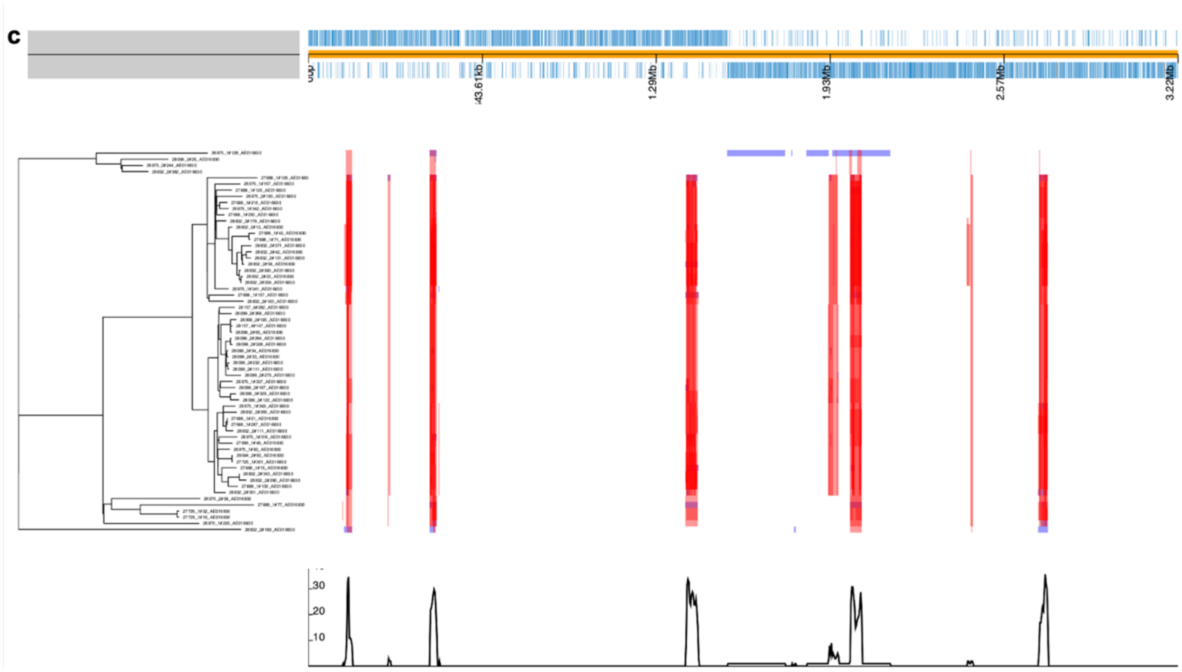

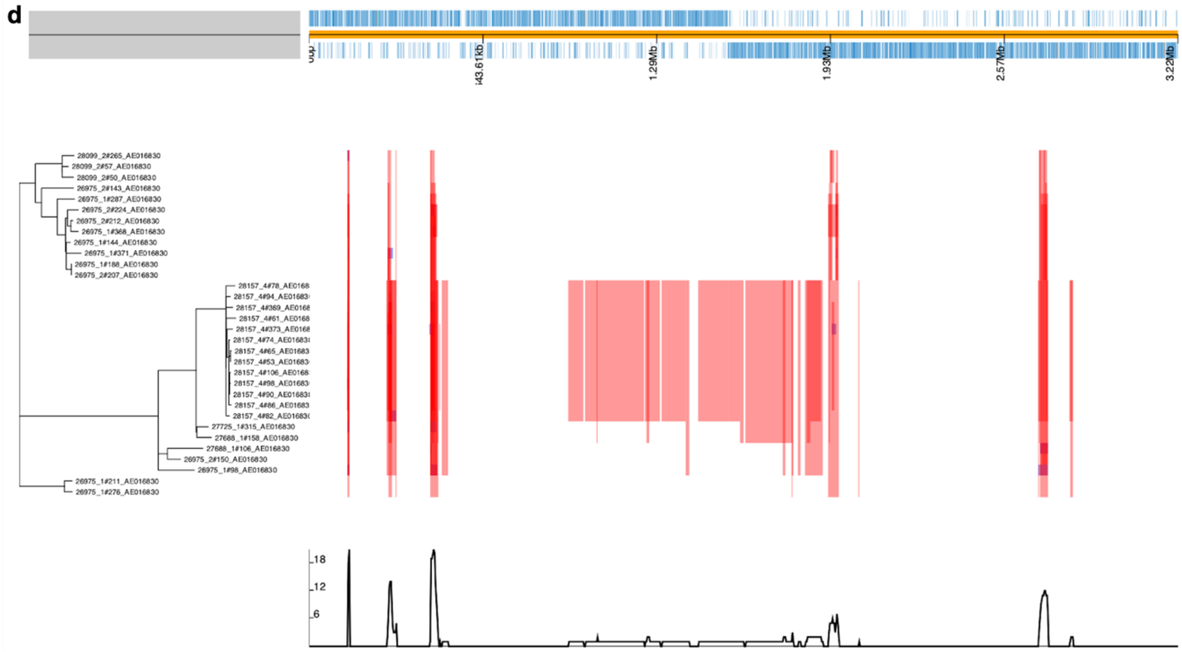

Supplementary Fig. 12: Recombination predictions by using Genealogies Unbiased By recomBinations In Nucleotide Sequences (Gubbins)[9], for hospital-associated (HA) Population Partitioning Using Nucleotide K-mers (PopPUNK)[7] clusters (PP). a, PP2 (n = 193). **b,** PP6 (n = 97). **c,** PP7 (n = 62). **d,** PP18 (n = 32). Alignments on *E. faecalis* V583. Recombination blocks across maximum-likelihood (ML) cluster phylogeny are depicted by using Phandango[10]: red colouring indicates ancestral blocks (occurring at a non-terminal

node), while blue indicates single isolates only. Reference genome annotation panel above the recombination blocks shows the linearised genome, with genes depicted as blue rectangles. Number of recombination events is plotted underneath the recombination blocks.

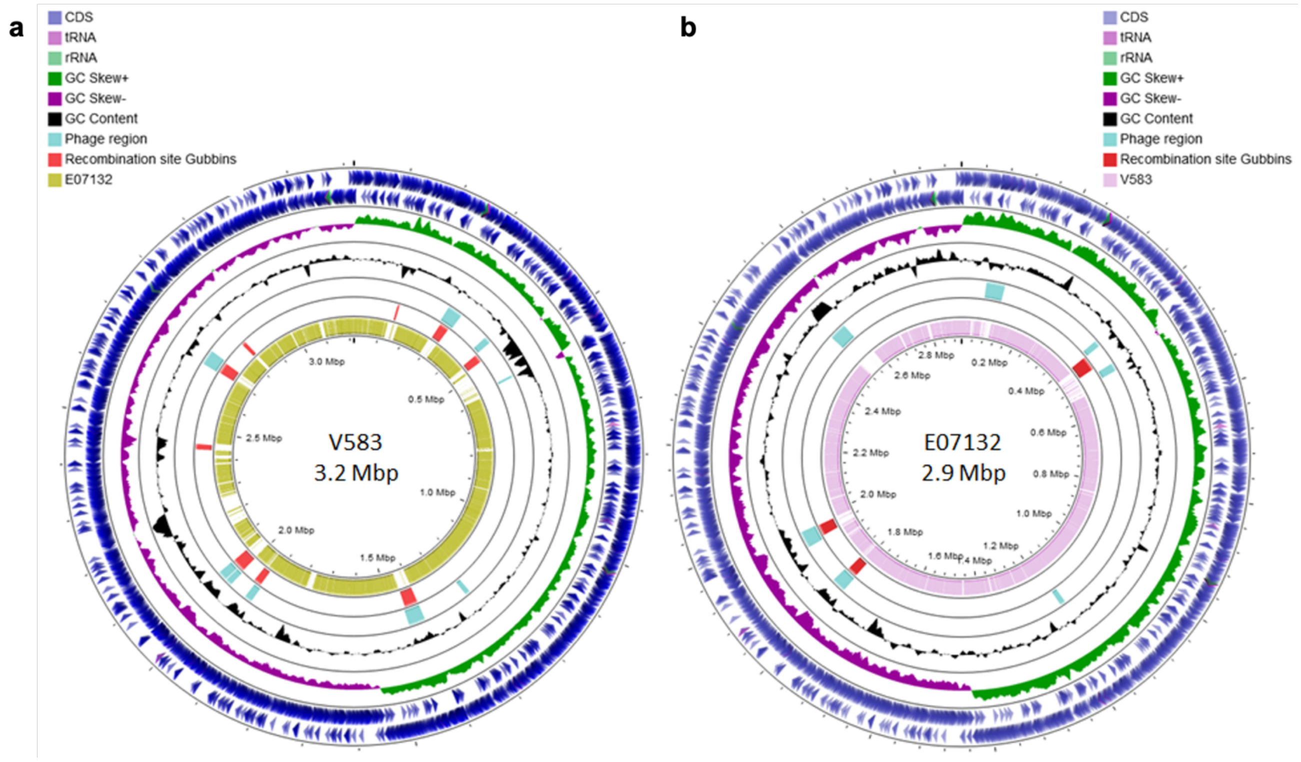

Supplementary Fig. 13: Genomic maps of V583 (a) and E07132 (b) used as references in the Genealogies Unbiased By recomBinations In Nucleotide Sequences (Gubbins)[9] analysis. Coloured as indicated in the legends, rings from outside to inside: CDS (blue), tRNA (pink), and rRNA (light green) genes; GC Skew + (green)/- (purple); GC content (black); phage integration sites as determined by Phaster[11] (light blue); recombination sites as determined by Gubbins[9] (red); and BLAST of the E07132 (ST97) against V583 (**a**) and V583 (ST6) against E07132 (**b**). White blocks indicate absence of genes.

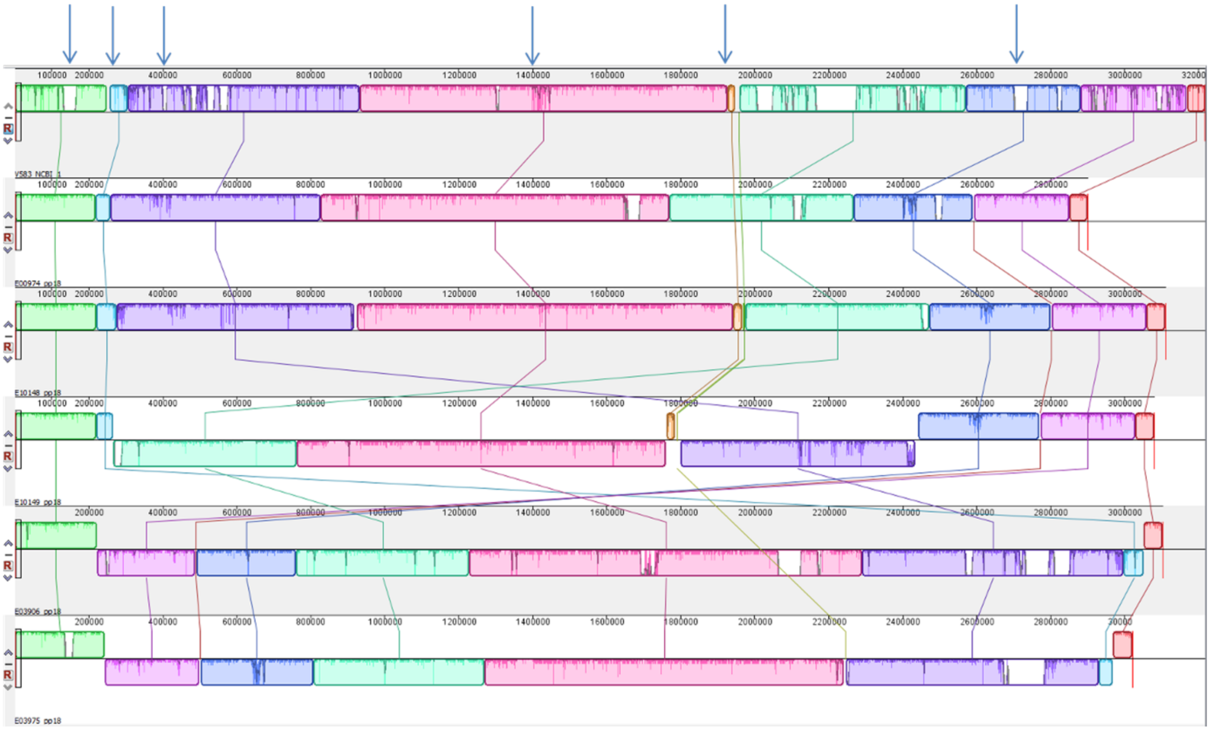

Supplementary Fig. 14: MAUVE[8] analysis on genome comparison of hospital-associated (HA) PP18 isolates. Coloured blocks indicating genomic rearrangements, and blue arrows indicating recombination peaks as determined by Genealogies Unbiased By recomBinations In Nucleotide Sequences (Gubbins)[9].

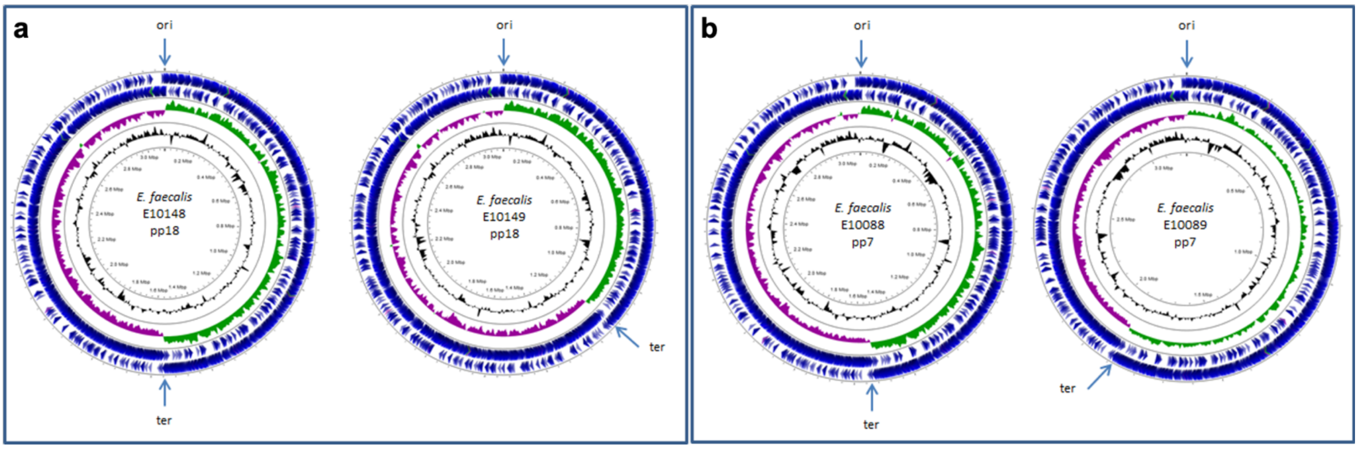

Supplementary Fig. 15: Genomic maps of PP18 (panel a) and PP7 (panel b) strains depicting a balanced replichore (left in panel) and replichore imbalance (right in panel). Rings from outside to inside: CDS (blue), tRNA (pink), and rRNA (light green) genes; GC Skew + (green)/- (purple); GC Content (black). Arrows indicate the ori and ter sites.

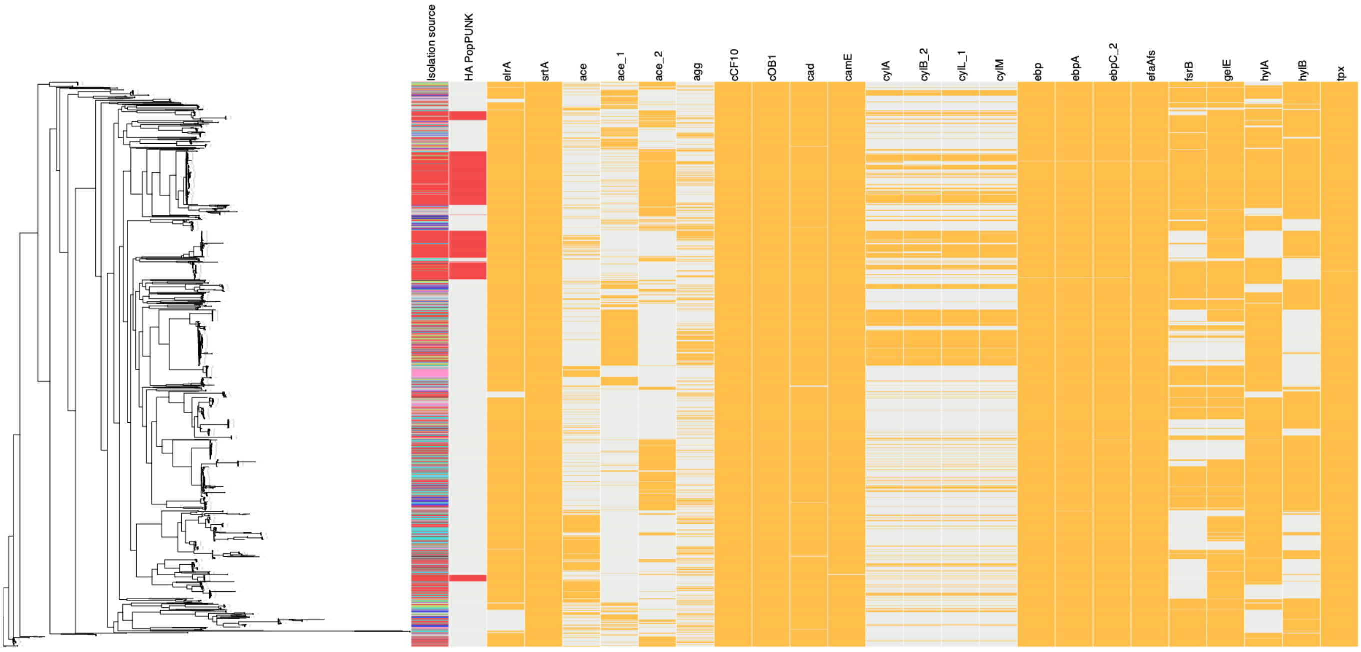

Supplementary Fig. 16. Collection-wide virulence gene patterns of *E. faecalis* isolates (*n* = 2,027) depict abundance of virulence genes but limited association with hospital isolates. First column, isolation sources: hospitalised patient (red), wild bird (dark blue), non-hospitalised person (light blue), old human isolates (black), environment (green), farm animal (pink), and others (grey). Second column, hospital-associated (HA) Population Partitioning Using Nucleotide K-mers (PopPUNK)[7] clusters (red). Presence (orange) and absence (grey) of virulence genes, as defined by using Antimicrobial Resistance Identification By Assembly (ARIBA)[12] against VirulenceFinder 2.0 database[13], is aligned with the species-wide reference mapping-based maximum likelihood (ML) phylogeny and depicted by using Phandango[10].

Supplementary Table 1. Analysing temporal signal and dating of hospital-associated (HA) Population Partitioning Using Nucleotide K-mers (PopPUNK) v.1.2.2[7] clusters by using TempEst v.1.5.3[14], Least-squares dating (LSD) v.0.3beta[15], and Bayesian Evolutionary Analysis by Sampling Trees (BEAST2) v.2.5.0[16–18].

HA PopPUNK cluster	TempEst				LSD		BEAST2[a]					
							Constant tree			Exponential tree		
	tMRCA[b]	Slope (rate)[c]	Correlation coefficient	R^2	tMRCA[b]	Rate[c]	tMRCA[b]	95% HPD[d] interval	clockRate[e]	tMRCA[b]	95% HPD[d] interval	clockRate[e]
PP2	1868	2.9391	0.5432	0.2950	1804	1.147	1846	1823–1866	5.719E-4	1844	1822–1865	5.635E-4
PP6	1987	9.4223	0.8512	0.7245	1965	4.200	1967	1961–1973	1.975E-3	1967	1961–1973	1.967E-3
PP7[f]	1928	5.2709	0.5887	0.3466	-	-	-	-	-	-	-	-
PP18	1959	5.1903	0.9385	0.8808	1917	2.645	1917	1891–1941	2.847E-3	1921	1896–1943	2.948E-3
PP20[g]	1637	1.1303	0.4487	0.2014	1542	0.958	NA	NA	NA	NA	NA	NA

[a] Three replicate BEAST2 runs combined by using LogCombiner v.2.5.1.
[b] tMRCA, the most recent common ancestor.
[c] Rate (TempEst, LSD), estimate of the rate of evolution in substitutions per site per year.
[d] HPD, highest posterior density.
[e] clockRate (BEAST2), rate of evolution averaged over the whole tree and all sites.
[f] PP7, TempEst on a subcluster of 55 isolates; LSD and BEAST2 on PP7 failed.
[g] PP20 excluded from BEAST2 dating analyses due to poor temporal signal.

References

1. Argimón, S. *et al.* Microreact: visualizing and sharing data for genomic epidemiology and phylogeography. *Microb Genom* **2**, e000093 (2016).
2. Tonkin-Hill, G. *et al.* Producing polished prokaryotic pangenomes with the Panaroo pipeline. *Genome Biol.* **21**, 180 (2020).
3. Shannon, P. *et al.* Cytoscape: a software environment for integrated models of biomolecular interaction networks. *Genome Res.* **13**, 2498–2504 (2003).
4. Arredondo-Alonso, S. *et al.* Plasmids Shaped the Recent Emergence of the Major Nosocomial Pathogen Enterococcus faecium. *MBio* **11**, (2020).
5. Arredondo-Alonso, S. *et al.* mlplasmids: a user-friendly tool to predict plasmid-and chromosome-derived sequences for single species. *Microbial genomics* **4**, (2018).
6. Katz, L. S. *et al.* Mashtree: a rapid comparison of whole genome sequence files. *Journal of Open Source Software* **4**, 1762 (2019).
7. Lees, J. A. *et al.* Fast and flexible bacterial genomic epidemiology with PopPUNK. *Genome Res.* **29**, 304–316 (2019).
8. Darling, A. E., Mau, B. & Perna, N. T. progressiveMauve: multiple genome alignment with gene gain, loss and rearrangement. *PLoS One* **5**, e11147 (2010).
9. Croucher, N. J. *et al.* Rapid phylogenetic analysis of large samples of recombinant bacterial whole genome sequences using Gubbins. *Nucleic Acids Res.* **43**, e15 (2015).
10. Hadfield, J. *et al.* Phandango: an interactive viewer for bacterial population genomics. *Bioinformatics* **34**, 292–293 (2018).
11. Arndt, D. *et al.* PHASTER: a better, faster version of the PHAST phage search tool. *Nucleic Acids Res.* **44**, W16–21 (2016).

12. Hunt, M. *et al.* ARIBA: rapid antimicrobial resistance genotyping directly from sequencing reads. *Microbial Genomics* vol. 3 (2017).

13. Joensen, K. G. *et al.* Real-time whole-genome sequencing for routine typing, surveillance, and outbreak detection of verotoxigenic Escherichia coli. *J. Clin. Microbiol.* **52**, 1501–1510 (2014).

14. Rambaut, A., Lam, T. T., Max Carvalho, L. & Pybus, O. G. Exploring the temporal structure of heterochronous sequences using TempEst (formerly Path-O-Gen). *Virus Evol* **2**, vew007 (2016).

15. To, T.-H., Jung, M., Lycett, S. & Gascuel, O. Fast Dating Using Least-Squares Criteria and Algorithms. *Syst. Biol.* **65**, 82–97 (2016).

16. Drummond, A. J. & Rambaut, A. BEAST: Bayesian evolutionary analysis by sampling trees. *BMC Evolutionary Biology* vol. 7 214 (2007).

17. Bouckaert, R. *et al.* BEAST 2: a software platform for Bayesian evolutionary analysis. *PLoS Comput. Biol.* **10**, e1003537 (2014).

18. Bouckaert, R. *et al.* BEAST 2.5: An advanced software platform for Bayesian evolutionary analysis. *PLoS Comput. Biol.* **15**, e1006650 (2019).
