# Supplementary material for: Apparent nosocomial adaptation of Enterococcus faecalis predates the modern hospital era
Source: Nat Commun. 2021 Mar 9;12:1523. doi: 10.1038/s41467-021-21749-5 (PMC7943827; doi:10.1038/s41467-021-21749-5)
Supplement: Supplementary file 3 — Descriptions of Additional Supplementary Files [file 41467_2021_21749_MOESM3_ESM.pdf]

Descriptions of Additional Supplementary Files

Supplementary Data 1

Description: Gene content comparison of hospital-associated clusters and commensal isolates of *E. faecalis* using logistic regression analysis and Bonferroni correction.

Supplementary Data 2

Description: Collection-wide screening of antimicrobial resistance and virulence genes in *E. faecalis*.
