# Supplementary material for: Apparent nosocomial adaptation of Enterococcus faecalis predates the modern hospital era
Source: Nat Commun. 2021 Mar 9;12:1523. doi: 10.1038/s41467-021-21749-5 (PMC7943827; doi:10.1038/s41467-021-21749-5)
Supplement: Supplementary file 6 — Reporting summary [file 41467_2021_21749_MOESM6_ESM.pdf]

nature research

Corresponding author(s): Anna K. Pöntinen, Jukka Corander

Last updated by author(s): Jan 27, 2021

Reporting Summary

Nature Research wishes to improve the reproducibility of the work that we publish. This form provides structure for consistency and transparency in reporting. For further information on Nature Research policies, see our Editorial Policies and the Editorial Policy Checklist.

Statistics

For all statistical analyses, confirm that the following items are present in the figure legend, table legend, main text, or Methods section.

n/a	Confirmed	
☐	☒	The exact sample size (*n*) for each experimental group/condition, given as a discrete number and unit of measurement
☐	☒	A statement on whether measurements were taken from distinct samples or whether the same sample was measured repeatedly
☐	☒	The statistical test(s) used AND whether they are one- or two-sided<br>*Only common tests should be described solely by name; describe more complex techniques in the Methods section.*
☐	☒	A description of all covariates tested
☐	☒	A description of any assumptions or corrections, such as tests of normality and adjustment for multiple comparisons
☐	☒	A full description of the statistical parameters including central tendency (e.g. means) or other basic estimates (e.g. regression coefficient) AND variation (e.g. standard deviation) or associated estimates of uncertainty (e.g. confidence intervals)
☐	☒	For null hypothesis testing, the test statistic (e.g. *F*, *t*, *r*) with confidence intervals, effect sizes, degrees of freedom and *P* value noted<br>*Give P values as exact values whenever suitable.*
☐	☒	For Bayesian analysis, information on the choice of priors and Markov chain Monte Carlo settings
☒	☐	For hierarchical and complex designs, identification of the appropriate level for tests and full reporting of outcomes
☒	☐	Estimates of effect sizes (e.g. Cohen's *d*, Pearson's *r*), indicating how they were calculated

Our web collection on statistics for biologists contains articles on many of the points above.

Software and code

Policy information about availability of computer code

Data collection	No software was used for data collection.
Data analysis	Hybrid assemblies were performed with Unicycler v.0.4.7 (https://github.com/rrwick/Unicycler). Bioawk v.20110810 (https://github.com/lh3/bioawk) was used to compute the genome size of each isolate, Filtlong v.0.2.0 (https://github.com/rrwick/Filtlong) to estimate ONT read quality, and Bandage v.0.8.1 (https://github.com/rrwick/Bandage) to retrieve the genome assembly statistics. The hybrid assembly pipeline is publicly available at https://github.com/arredondo23/hybrid_assembly_slurm. Maximum likelihood phylogeny was inferred using RaxML v.8.2.8. Collection-wide whole-genome clustering was defined using Population Partitioning Using Nucleotide K-mers (PopPUNK) v.1.2.2 and Pangenome Neighbour Identification for Bacterial Populations (PANINI) (https://panini.pathogen.watch/), and cluster-specific recombination was identified and removed using Genealogies Unbiased By recomBinations In Nucleotide Sequences (Gubbins) v.2.4.0 (https://github.com/sanger-pathogens/gubbins). Pangenomes were estimated using Panaroo v.1.2.0 (https://gtonkinhill.github.io/panaroo/#/). Plasmid predictions were performed using mlplasmids (mlplasmids (https://gitlab.com/sirarredondo/mlplasmids). Cluster-specific phylogeny dating was estimated and analysed using TempEst v.1.5.3, least-squares dating (LSD) v.0.3beta, Bayesian Evolutionary Analysis by Sampling Trees (BEAST2) v.2.5.0, Traces v.1.7.1, LogCombiner v.2.5.1, and TreeAnnotator v.2.5.1. Geneious software package (Biomatters, Ltd., Auckland, New Zealand) was used in analysing cluster-specific genomic reorganisation. Resistance and virulence genes were screened using Antimicrobial Resistance Identification By Assembly (ARIBA) v.2.14.4 and ABRicate v.0.9.8. FigTree v.1.4.4, Phandango (https://jameshadfield.github.io/phandango/#/), CGView Comparison Tool (https://github.com/paulstothard/cgview_comparison_tool), R v.3.6.2 and v.4.0.2, Cytoscape (https://cytoscape.org), and Microreact (https://microreact.org/) were used in visualising the results. Custom codes generated within the study for plasmid analyses (https://gitlab.com/sirarredondo/efaecalis_plasmids), gene content comparison and accessory genome network analyses (https://github.com/gtonkinhill/Efcm_Efcs_analysis), and empirical cumulative distribution functions and permutation tests (https://github.com/akpontinen/Efaecalis_eCDF) are publicly available at the respective community repositories.

For manuscripts utilizing custom algorithms or software that are central to the research but not yet described in published literature, software must be made available to editors and reviewers. We strongly encourage code deposition in a community repository (e.g. GitHub). See the Nature Research guidelines for submitting code & software for further information.

Data

Policy information about availability of data

All manuscripts must include a data availability statement. This statement should provide the following information, where applicable:

- Accession codes, unique identifiers, or web links for publicly available datasets
- A list of figures that have associated raw data
- A description of any restrictions on data availability

Illumina fastq reads of the E. faecalis isolates are publicly available at the ENA project “PRJEB28327 [https://www.ebi.ac.uk/ena/browser/view/PRJEB28327]” and long-read ONT sequences and hybrid assemblies at the ENA project “PRJEB40976 [https://www.ebi.ac.uk/ena/browser/view/PRJEB40976]”. Descriptive data on the collection of 2,027 E. faecalis isolates, together with ML phylogeny, PANINI network on Panaroo pangenome output, and temporal and geographic metadata is available within the public Microreact project https://microreact.org/project/3T9X5PlUD. Phylogeny and descriptive data on the old isolate plasmids from 1943 to 1985 are available within the public Microreact project https://microreact.org/project/oR27udmSsi96yeLmL41Wdg. E. faecium Illumina reads were retrieved from the ENA project “PRJEB28495 [https://www.ebi.ac.uk/ena/browser/view/PRJEB28495]”. All supporting accession codes are available within the article. Source data are provided with this paper, within the custom code repositories detailed in Code availability, and within the Microreact projects.

Field-specific reporting

Please select the one below that is the best fit for your research. If you are not sure, read the appropriate sections before making your selection.

☒ Life sciences ☐ Behavioural & social sciences ☐ Ecological, evolutionary & environmental sciences

For a reference copy of the document with all sections, see nature.com/documents/nr-reporting-summary-flat.pdf

Life sciences study design

All studies must disclose on these points even when the disclosure is negative.

Sample size	In total, 2,027 E. faecalis isolates were used in this study. The total study collection was aimed to cover a large set of clinical human samples, with approximately half of the total samples isolated from hospitalised patients (n = 967), in addition to non-hospitalised persons (n = 391). To additionally cover a wide range of non-human sources, environmental sites (n =156), farm animals (n = 130), and a variety of others (n = 219), such as food samples and isolates from pet animals and wild mammals, were included. A large set of wild bird isolates (n = 136) was deemed an important asset in investigating in more detail the transmission of antimicrobial resistance genes within migratory birds. The collection was also designed to cover a wide geographic range, with isolates from 24 countries, and a wide temporal range, with isolates from 1936 to 2018. Specifically, the collection included a subset of old human isolates (n= 28), some of which preceding the modern antibiotic era. Statistical sample size calculations were not performed as the total number of isolates subjected to whole-genome sequencing was determined by isolate availability, laboratory resources and amount of funding available.
Data exclusions	One isolate out of 2,027 failed the assembly and annotation pipeline and was, thus, excluded from the analyses run on assemblies; this isolate was included in analyses run on sequence reads. Seven isolates lacking plasmids were accordingly excluded from the plasmidome evolutionary analyses of the old isolates, while included elsewhere in the study. Strains with unknown isolation dates were removed from the dating analyses, and PopPUNK cluster 20 was removed from BEAST2 dating analysis due to poor temporal signal, as determined by TempEst. Virulence gene hylEfm was disregarded from E. faecalis virulence gene analysis as specific to E. faecium.
Replication	Where needed, experiments were replicated successfully. In phylogeny dating analysis, BEAST2 analyses were performed for 100,000,000 generations sampled every 1,000 states and three replicate runs per each cluster and tree model. In comparing the empirical cumulative distribution functions between isolates from different sources, 10,000 random permutations were generated.
Randomization	Randomization was not used since we sequenced all old isolates available in the study laboratories and selected modern isolates based on a representative set of their metadata characteristics, including year of isolation, source and country of origin.
Blinding	Blinding was not relevant, since this is not an interventional study.

Reporting for specific materials, systems and methods

We require information from authors about some types of materials, experimental systems and methods used in many studies. Here, indicate whether each material, system or method listed is relevant to your study. If you are not sure if a list item applies to your research, read the appropriate section before selecting a response.

Materials & experimental systems

n/a	Involved in the study
☒	☐ Antibodies
☒	☐ Eukaryotic cell lines
☒	☐ Palaeontology and archaeology
☒	☐ Animals and other organisms
☒	☐ Human research participants
☒	☐ Clinical data
☒	☐ Dual use research of concern

Methods

n/a	Involved in the study
☒	☐ ChIP-seq
☒	☐ Flow cytometry
☒	☐ MRI-based neuroimaging
